# Volatiles of Black Pepper Fruits (*Piper nigrum* L.)

**DOI:** 10.3390/molecules24234244

**Published:** 2019-11-21

**Authors:** Noura S. Dosoky, Prabodh Satyal, Luccas M. Barata, Joyce Kelly R. da Silva, William N. Setzer

**Affiliations:** 1 Aromatic Plant Research Center, Suite 100, Lehi, UT 84043, USA; ndosoky@aromaticplant.org (N.S.D.); psatyal@aromaticplant.org (P.S.); 2 Programa de Pós-Graduação em Biotecnologia, Universidade Federal do Pará, Belém 66075-110, PA, Brazil; luccas.miranda2@gmail.com (L.M.B.); joycekellys@ufpa.br (J.K.R.d.S.); 3 Department of Chemistry, University of Alabama in Huntsville, Huntsville, AL 35899, USA

**Keywords:** *Piper nigrum*, black pepper, essential oil composition, cluster analysis

## Abstract

Black pepper (*Piper nigrum*) is historically one of the most important spices and herbal medicines, and is now cultivated in tropical regions worldwide. The essential oil of black pepper fruits has shown a myriad of biological activities and is a commercially important commodity. In this work, five black pepper essential oils from eastern coastal region of Madagascar and six black pepper essential oils from the Amazon region of Brazil were obtained by hydrodistillation and analyzed by gas chromatography-mass spectrometry. The major components of the essential oils were α-pinene, sabinene, β-pinene, δ-3-carene, limonene, and β-caryophyllene. A comparison of the Madagascar and Brazilian essential oils with black pepper essential oils from various geographical regions reported in the literature was carried out. A hierarchical cluster analysis using the data obtained in this study and those reported in the literature revealed four clearly defined clusters based on the relative concentrations of the major components.

## 1. Introduction

Genus *Piper* (Piperaceae) is represented by about 1500–2000 species of perennial evergreen climbing, lianescent herbs or shrubs distributed in tropical and subtropical regions. Pepper (*Piper nigrum* L.) is one of the oldest and most extensively used spices and traditional medicines known to mankind. The plant is believed to have originated in India and Indonesia, and has been cultivated throughout the tropical regions [1,2,3]. India, Brazil, Indonesia, Malaysia, Vietnam, and Sri Lanka are the major countries of *P. nigrum* production [3,4]. The plant can reach up to 50–60 cm in height [5] and is characterized by its simple, alternate leaves, with a few rare cases of opposite or verticillate leaves [1]. The most commonly used part of the plant is the aromatic fruit. Interestingly, white, green, and black peppers are products of the *P. nigrum* fruits at different ripening stages [3]. White pepper is obtained from the fully ripened fruits after removing the outer skin, green pepper is the unripe fruits, and black pepper is collected before full maturity of the fruit [1,3]. Black pepper has a stronger flavor compared to white pepper while green pepper is characterized by its fresh and herbal flavor. The alkaloid piperine is responsible for the pungent flavor of black pepper [3].

*P. nigrum* is well-known for its medicinal properties. Traditionally, it has been used in many Asian countries for treating indigestion, asthma, pain, respiratory tract infections, and rheumatoid arthritis [6]. It is also a stimulant, digestive, tonic, and antiseptic [5]. Black pepper essential oil (EO) showed antioxidant, carminative, larvicidal, antibacterial, and antifungal activities [2,7,8]. *P. nigrum* oil showed strong antibacterial activity against *Acinetobacter calcoacetica*, *Alcaligenes faecalis*, *Bacillus subtilis*, *Beneckea natriegens*, *Brevibacterium linens*, *Brocothrix thermosphacta*, *Citrobacter freundii*, *Clostridium sporogenes*, *Enterococcus faecalis*, *Erwinia carotovora*, *Escherichia coli*, *Flavobacterium suaveolens*, *Leuconostoc cremoris*, *Micrococcus luteus*, *Moraxella* sp., *Proteus vulgaris*, *Pseudomonas aeruginosa*, *Salmonella pullorum*, *Serratia marcescens*, *Staphylococcus aureus*, and *Yersinia enterocolitica* [9]. In addition, black pepper EO inhibited *Staphylococcus aureus* biofilm formation via down-regulating the expressions of the α-toxin gene (hla), the nuclease genes, and the regulatory genes [10]. It was also reported to decrease *S. aureus* virulence in *Caenorhabditis elegans* [10]. The oil prevented the formation of aflatoxin B1-DNA adduct in a microsomal enzyme-mediated reaction (in vitro) [11]. *P. nigrum* fruit oil showed some insecticide activity (contact toxicity) against *Sitophilus zeamais* (LD_50_ = 26.4 ± 1.5 µL/g) [1]. In patients with poor vein visibility, topical application of black pepper EO (20% in aloe vera gel) was reported to enhance vein visibility and intravenous catheter insertion [12]. Inhalation of black pepper EO was able to activate the insular or orbitofrontal cortex, which led to improved reflexive swallowing movement in older post-stroke patients with swallowing dysfunction (dysphagia) in a one-month randomized, controlled study [13]. Moreover, inhaling *P. nigrum* oil was effective in reducing smoking withdrawal symptoms including cigarette craving and anxiety [14]. Inhalation of a single drop of black pepper EO on a tissue for two minutes when craving nicotine resulted in reduced nicotine craving and increased delay time before the next tobacco use [15]. In combination with a massage, the oil can be used as a preventive treatment for cutaneous wrinkling and ageing via penetrating the skin and effectively inhibiting the activity of elastase (enzyme that degenerates dermal elastin) [16]. Compared to butylated hydroxyanisole (BHA) and butylated hydroxytoluene (BHT), *P. nigrum* EO and extracts were reported to have strong in vitro and in vivo antioxidant and radical scavenging activities [2,5,8]. Oral administration of the oil for a month to mice, considerably reduced the production of super oxide radicals and increased the blood levels of superoxide dismutase, glutathione, and glutathione reductase as well as the liver levels of catalase, superoxide dismutase, glutathione, glutathione-*S*-transferase, and glutathione peroxidase [8]. Intraperitoneal administration of black pepper EO (500 mg/kg body weight) for five consecutive days showed strong anti-inflammatory and anti-nociceptive properties in Balb/C mice [8]. The oil inhibited the carrageenan-induced and dextran-induced acute inflammation and the formalin-induced chronic inflammation. *P. nigrum* extracts inhibited the production of pro-inflammatory nuclear factor (NF-κB), cyclooxygenase-1 (COX-1) and cyclooxygenase-2 (COX-2), and tumor cell proliferation [17]. In another randomized, double-blind, placebo-controlled study for nine weeks, inhalation of *P. nigrum* EO for 15 min showed significant analgesic activities in 54 patients with different pain types [18].

Due to the high economical and medicinal value of *P. nigrum*, it was subjected to several phytochemical studies [1,3]. Black pepper oil, which is responsible for its characteristic flavor and aroma, accounts for about 3–6% [3,8]. It ranges from colorless to greenish in color, with a spicy (peppery) scent. The oil is usually obtained from *P. nigrum* fruits by distillation, simultaneous distillation-extraction (SDE), solid phase microextraction (SPME), or supercritical fluid extraction [3,19,20]. More than a hundred compounds have been reported in black pepper oil. The oil is dominated by monoterpene hydrocarbons (47–64%) followed by sesquiterpene hydrocarbons (30–47%) [21]. In the literature, the main components frequently mentioned in *P. nigrum* oils seem to be β-caryophyllene, limonene, β-pinene, α-pinene, δ-3-carene, sabinene, and myrcene with great variations in their percentages. These variations could be attributed to differences in environmental factors, plant variety, cultivation practices, harvesting stage, and method of extraction. It may be worth mentioning that storage of ground black, green, and white pepper affects the oil composition. In the current study, we investigated the composition of the essential oils of *Piper nigrum* from the APRC collection from Madagascan east coastal region as well as essential oils from several cultivars cultivated in Pará State, Brazil.

## 2. Results and Discussion

Five *Piper nigrum* essential oils from a collection of oils from the Madagascan east coastal region, deposited with the Aromatic Plant Research Center (APRC) collection, were analyzed by gas chromatography–mass spectrometry (GC-MS). A total of 78 compounds were identified accounting for more than 99% of the compositions. The oils were mainly made of monoterpene hydrocarbons (59.2–80.1%) and sesquiterpene hydrocarbons (17.0–37.7%) while oxygenated terpenoids accounted for about 1.3–2.7%. The major components were α-pinene (5.1–28.7%), β-caryophyllene (8.7–25.6%), limonene (15.1–19.5%), β-pinene (9.1–15.3%), and δ-3-carene (9.0–12.8%; Table 1).

Six different cultivars (“Bragantina”, “Cingapura”, “Clonada”, “Equador”, “Guajarina”, and “Uthirankota”) of *P. nigrum*, cultivated in Pará State, Brazil, were collected, hydrodistilled, and analyzed (Table 2). The oil yields (%) calculated for the cultivars (“Bragantina”, “Cingapura”, “Clonada”, “Equador”, “Guajarina”, and “Uthirankota” were 0.86%, 0.21%, 0.85%, 0.64%, 1.49%, and 1.06%, respectively.

These *P. nigrum* fruit essential oils were also rich in monoterpene hydrocarbons (76.6–89.5%) and sesquiterpene hydrocarbons (0.8–17.8%), but also had sizeable quantities of oxygenated monoterpenoids (2.2–8.2%), and oxygenated sesquiterpenoids (0.6–5.0%). The major components in the black pepper oils from Pará State were β-pinene (20.3–48.0%) and limonene (24.3–38.1%).

The oil compositions presented in this work show quantitative similarities and differences from previously published studies on black pepper oils. Bagheri and co-workers compared the composition of Malaysian pepper oils obtained by hydrodistillation and supercritical carbon dioxide extraction (SC-CO_2_). The hydrodistilled oil was made of β-caryophyllene (18.60%), limonene (14.95%), sabinene (13.19%), β-pinene (9.71%), δ-3-carene (8.56%), and α-pinene (7.96%) while the SC-CO_2_ oil had β-caryophyllene (25.38%), limonene (15.64%), sabinene (13.63%), δ-3-carene (9.34%), and β-pinene (7.27%) [5]. The major components in black pepper corn oils of Malaysian origin extracted by simultaneous distillation and extraction (SDE) were limonene (23.9–29.7%), β-pinene (15.6–19.0%), β-caryophyllene (10.3–14.0%), δ-3-carene (8.7–10.6%), and α-pinene (6.6–7.3%) while the ground pepper oil had β-caryophyllene (38.1–63.0%), limonene (3.0–14.3%), δ-3-carene (3.0%–13.8%), and β-pinene (1.5–5.9%) [3].

Hydrodistillation of *P. nigrum* fruits grown in Cameroon produced an oil made of δ-3-carene (18.5%), limonene (14.7%), β-caryophyllene (12.8%), sabinene (11.2%), α-pinene (5.6%), and β-pinene (6.7%) [1]. α-Pinene (25.4%), limonene (21.0%), β-pinene (15.7%), and δ-3-carene (10.8%) were reported as the main constituents of *P. nigrum* fruit oil from Madagascar [24]. Interestingly, Chinese pepper EO obtained by microwave distillation and headspace solid-phase microextraction (MD-HS-SPME) has shown β-caryophyllene (23.49%), δ-3-carene (22.20%), limonene (18.68%), and β-pinene (8.92%) as the main constituents [19]. A steam-distilled oil from India contained β-caryophyllene (23.98%), limonene (14.36%), α-terpinene (13.26%), caryophyllene oxide (8.04%), and α-pinene (5.0%) [8] while a hydrodistilled sample from India had β-caryophyllene (29.9%), limonene (13.2%), β-pinene (7.9%), and sabinene (5.9%) [2]. Martins and co-workers obtained *P. nigrum* EO from S. Tome e Principe (hydrodistillation) and found limonene (18.8%), sabinene (16.5%), β-caryophyllene (15.1%), β-pinene (10.7%), and α-pinene (5.7%) as the main constituents [24]. The oil obtained from ground black pepper (supercritical fluid extraction, USA) was made of β-caryophyllene (21.77%), limonene (19.82%), δ-3-carene (14.34%), sabinene (11.64%), and myrcene (7.70%) [20]. Several authors have analyzed various geographical varieties of essential oils of black pepper including Thevanmundi [25], Poonjaranmuna [25], Valiakaniakadan [25], Subhakara [25], Sreekara [26], Kuching [26], Vellanamban [26], Aimpiriyan [27], Narayakodi [27], Neelamundi [27], Uthirankotta [27], and Panniyur [28].

**Figure 1:**
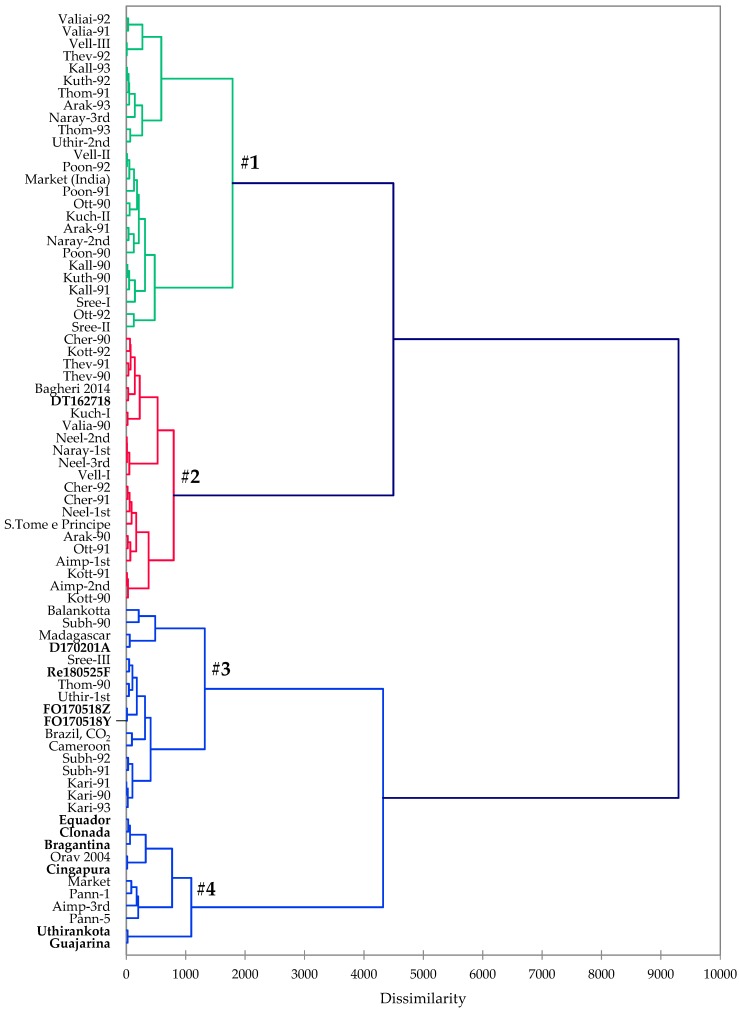
Dendrogram obtained from the agglomerative hierarchical cluster analysis of *Piper nigrum* oils. Entries in bold font are from this study.

Based on *P. nigrum* essential oil compositions, a hierarchical cluster analysis of the oils from this work (five samples from Madagascar and six samples from Pará state, Brazil) and those reported in the literature (65 samples, Table 3) was carried out. The cluster analysis revealed four clearly defined clusters (Figure 1). The cluster centroids of the major components of *P. nigrum* oils are summarized in Table 4, illustrating the chemical differences in the three classes: Class #1 (β-caryophyllene > limonene > β-pinene > sabinene > α-pinene), Class #2 (β-caryophyllene > sabinene > limonene > β-pinene > α-pinene), Class #3 (limonene > β-caryophyllene > δ-3-carene > α-pinene > β-pinene), and Class 4 (β-pinene ≈ limonene > β-caryophyllene > α-pinene > myrcene). The first cluster was the largest representing 26 samples. APRC sample DT162718 fell in the second cluster that represents 22 samples while D170201A, FO170518Y, FO170518Z, and Re180525F fell in the third cluster that represents 17 samples. All of the Belem cultivar samples fell into the fourth cluster of 11 samples.

## 3. Materials and Methods

### 3.1. Essential Oils

Volatile oils from commercial suppliers were obtained from the collections of the Aromatic Plant Research Center (APRC, Lehi, UT, USA). The samples from Pará state were provided by EMBRAPA Amazônia Oriental (Brazilian Agricultural Research Corporation) and obtained by hydrodistillation in a Clevenger apparatus (100 g, 3 h). The oils were dried over anhydrous sodium sulfate and their yields calculated from the dry weight of the plant material.

### 3.2. Gas Chromatographic-Mass Spectral Analysis

The essential oils obtained from APRC were analyzed by gas chromatography-mass spectrometry (GC-MS) using a Shimadzu GCMS-QP2010 Ultra operated in the electron impact (EI) mode (electron energy = 70 eV), scan range = 40–400 atomic mass units, scan rate = 3.0 scans/s, and GC-MS solution software. The GC column was a ZB-5 fused silica capillary column with a (5% phenyl)-polymethylsiloxane stationary phase and a film thickness of 0.25 μm, a length of 30 m, and an internal diameter of 0.25 mm. The carrier gas was helium with a column head pressure of 552 kPa and flow rate of 1.37 mL/min. The injector temperature was 250 °C and the ion source temperature was 200 °C. The GC oven temperature was programmed for 50 °C initial temperature, then temperature was increased at a rate of 2 °C/min to 260 °C. A 7% *w*/*v* solution of the sample was prepared in dichloromethane and 0.1 μL was injected with a splitting mode (30:1).

Qualitative analysis of the Belém samples was carried out by gas chromatography-mass spectrometry (GC-MS) (Shimadzu QP2010 plus instrument, Shimadzu Scientific Instruments, Columbia, MD, USA) under the following conditions: Rtx-5MS silica capillary column (30 m × 0.25 mm film thickness, (Phenomenex, Torrance, CA, USA); programmed temperature, 60–240 °C (3 °C/min); injector temperature, 200 °C; carrier gas, helium, adjusted to a linear velocity of 1.2 mL/min; injection type, splitless; split flow was adjusted to yield a 20:1 ratio; septum sweep was a constant 10 mL/min; EIMS, electron energy, 70 eV; and temperature of the ion source and connection parts, 200 °C. The retention indices were calculated for all the volatile constituents using a homologous series of *n*-alkanes (C_8_–C_32_, Sigma-Aldrich). Identification of the oil components was based on their retention indices and by comparison of their mass spectral fragmentation patterns with those reported in the literature [23], and our own in-house library [24]. The component percentages are based on peak integrations without standardization.

### 3.3. Hierarchical Cluster Analysis

*P. nigrum* oils obtained from this work as well as the published literature were used in the cluster analysis. The essential oil compositions were treated as operational taxonomic units (OTUs), and the concentrations (percentages) of the major components (α-thujene, α-pinene, camphene, sabinene, β-pinene, myrcene, α-phellandrene, δ-3-carene, *p*-cymene, limonene, β-phellandrene, *(E)*-β-ocimene, terpinolene, linalool, δ-elemene, α-cubebene, α-copaene, β-elemene, β-caryophyllene, α-guaiene, α-humulene, germacrene D, β-selinene, α-selinene, α-farnesene, β-bisabolene, δ-cadinene, elemol, and caryophyllene oxide) were used to determine the chemical associations between the essential oils using agglomerative hierarchical cluster (AHC) analysis using XLSTAT Premium, version 2018.5.53172 (Addinsoft, Paris, France). Dissimilarity was determined using Euclidean distance, and clustering was defined using Ward’s method.

## 4. Conclusions

The essential oils of black pepper have been analyzed by GC-MS. The oils were dominated by monoterpene hydrocarbons. Black pepper oils from various geographical locations have shown qualitative similarities with differences in the concentrations of their major components. β-Caryophyllene, limonene, β-pinene, α-pinene, δ-3-carene, sabinene, and myrcene were the main components of *P. nigrum* oil. The cluster analysis revealed four clearly defined clusters for *P. nigrum*.

## Figures and Tables

**Table 1:** Chemical compositions of *Piper nigrum* fruit volatile oils from the Aromatic Plant Research Center (APRC) collection.

RI ^a^	RI ^b^	Compound	D170201A	FO170518Y	FO170518Z	Re180525F	DT162718
921	921	Tricyclene	---	---	---	---	tr ^c^
924	924	α-Thujene	0.1	0.1	0.2	0.9	0.6
932	932	α-Pinene	28.7	12.9	15.2	5.1	11.1
945	945	α-Fenchene	---	---	---	---	tr
954	946	Camphene	1.0	0.6	0.6	0.1	0.3
969	969	Sabinene	0.2	0.4	0.7	6.9	13.9
974	974	β-Pinene	15.3	12.6	13.4	9.1	15.1
988	988	Myrcene	2.6	2.7	3.0	2.1	1.3
1002	1002	α-Phellandrene	2.2	2.5	3.4	2.3	0.6
1003	1003	*p*-Mentha-1(7),8-diene	tr	---	---	---	---
1008	1008	δ-3-Carene	9.0	11.7	12.8	11.7	10.4
1017	1014	α-Terpinene	0.1	---	0.1	0.2	0.1
1020	1020	*p*-Cymene	0.3	0.8	0.4	1.1	0.3
1022	1022	*o*-Cymene	---	0.1	---	0.1	---
1025	1025	β-Phellandrene	0.2	---	---	1.4	0.9
1026	1026	1,8-Cineole	0.2	0.3	0.4	---	tr
1029	1024	Limonene	19.5	18.2	18.2	17.4	15.1
1032	1032	(*Z*)-β-Ocimene	tr	---	---	---	---
1044	1044	(*E*)-β-Ocimene	0.4	0.2	0.3	0.1	0.1
1054	1054	γ-Terpinene	0.1	0.1	0.1	0.3	0.1
1069	1065	*cis*-Sabinene hydrate	---	---	---	0.1	0.1
1086	1086	Terpinolene	0.5	0.8	0.9	0.5	0.2
1095	1095	Linalool	0.6	0.4	0.3	0.5	0.3
1100	1098	*trans*-Sabinene hydrate	---	---	---	---	0.1
1135	1135	*trans*-Pinocarveol	---	---	---	---	tr
1140	1140	*cis*-β-Terpineol	---	0.2	---	---	---
1141	1141	Camphor	0.1	0.1	tr	---	---
1174	1174	Terpinen-4-ol	0.1	---	---	0.3	0.2
1186	1186	α-Terpineol	0.3	---	0.2	---	0.1
1328	1330	Bicycloelemene	0.1	---	---	0.1	tr
1335	1335	δ-Elemene	1.1	2.9	2.9	0.9	1.0
1348	1345	α-Cubebene	tr	0.1	0.1	0.3	0.1
1369	1369	Cyclosativene ^d^	0.2	---	---	0.1	0.1
1373	1373	α-Ylangene	---	0.1	0.1	---	---
1374	1374	Isoledene	---	0.2	0.2	---	---
1376	1374	α-Copaene	0.1	0.2	0.2	3.1	2.0
1387	1387	β-Cubebene	---	---	---	0.2	0.2
1389	1389	β-Elemene	1.0	1.8	1.4	1.1	0.2
1408	1408	(*Z*)-β-Caryophyllene	---	---	---	---	tr
1409	1408	α-Gurjunene	0.1	0.1	0.1	0.2	tr
1417	1417	(*E*)-β-Caryophyllene	8.7	18.3	15.2	25.6	21.6
1430	1430	β-Copaene	---	0.1	---	0.1	0.1
1436	1434	γ-Elemene	0.1	0.1	0.1	---	---
1437	1437	α-Guaiene	0.1	0.6	0.3	0.4	tr
1448	1448	*cis*-Murrola-3,5-diene	---	---	---	---	tr
1452	1452	α-Humulene	0.9	1.8	1.3	1.6	0.7
1461	1461	*cis*-Cadina-1(6),4-diene	---	---	---	---	tr
1475	1475	*trans*-Cadina-1(6),4-diene	tr	0.1	0.4	---	tr
1484	1484	Germacrene D	2.0	2.5	3.0	0.2	0.1
1490	1489	β-Selinene	1.3	2.0	1.9	1.5	0.1
1493	1493	*trans*-Muurola-4(14),5-diene	---	---	---	---	0.1
1496	1496	Viridiflorene	---	---	---	0.1	---
1498	1498	α-Selinene	1.0	1.5	1.5	1.3	---
1500	1500	α-Muurolene	---	0.1	0.1	0.2	0.3
1500	1500	Bicyclogermacrene	---	---	---	---	0.3
1501	1501	*epi*-Zonarene	0.1	---	---	---	---
1505	1505	β-Bisabolene	tr	0.3	---	0.1	0.7
1505	1505	(*E*,*E*)-α-Farnesene	---	---	0.1	---	---
1514	1514	Cubebol	tr	---	---	0.3	0.1
1518	1521	α-Panasinsen	tr	0.1	0.1	---	---
1521	1521	*trans*-Calamenene	---	---	---	tr	tr
1523	1522	δ-Cadinene	0.1	0.1	0.2	0.8	0.7
1533	1533	*trans*-Cadina-1,4-diene	---	---	---	---	tr
1548	1548	α-Elemol	tr	---	---	---	---
1561	1559	Germacrene B	0.1	0.2	0.1	---	---
1561	1561	(*E*)-Nerolidol	---	---	---	---	tr
1577	1577	Spathulenol	tr	---	---	0.1	tr
1582	1582	Caryophyllene oxide	0.2	1.4	0.3	0.8	0.5
1608	1608	Humulene epoxide II	---	0.1	---	---	---
1618	1618	1,10-di-*epi*-Cubenol	0.1	---	---	---	---
1626	1629	*iso*-Spathulenol	0.3	---	---	0.3	0.1
1639	1644	*allo*-Aromadendrene epoxide	tr	---	---	---	---
1640	1640	τ-Muurolol	---	---	---	0.1	tr
1644	1644	α-Muurolol (=δ-Cadinol)	---	---	---	0.3	0.1
1651	1651	Pogostol	0.1	---	---	---	---
1652	1652	α-Cadinol	0.1	---	0.1	---	---
1660	1660	Selin-11-en-4α-ol	tr	---	---	---	---
1685	1685	Germacra-4(15),5,10(14)-trien-1α-ol	0.5	---	---	---	---
		Monoterpene hydrocarbons	80.1	63.5	69.2	59.2	69.9
		Oxygenated monoterpenoids	1.2	1.0	0.9	0.8	0.7
		Sesquiterpene hydrocarbons	17.0	33.3	29.2	37.7	28.2
		Oxygenated sesquiterpenoids	1.3	1.5	0.4	1.9	0.8
		Total identified	99.7	99.3	99.7	99.5	99.7

^a^ RI = “Retention Index” determined in reference to a homologous series of *n*-alkanes. ^b^ Retention indices from the databases [22,23]. ^c^ tr = “trace” (<0.05%). ^d^ This compound may be cyclocopacamphene, an epimer of cyclosativene.

**Table 2:** Chemical compositions of *Piper nigrum* fruit volatile oils cultivated in Pará State, Brazil.

RI ^a^	RI ^b^	Compound	Bragantina	Cingapura	Clonada	Equador	Guajarina	Uthirankota
921	924	α-Thujene	0.2	---	0.2	0.6	0.7	1.4
929	932	α-Pinene	9.2	6.8	8.0	7.4	11.3	10.3
965	969	Sabinene	---	0.1	0.5	---	---	---
973	974	β-Pinene	33.6	20.3	29.2	29.2	45.6	48.0
987	988	Myrcene	2.5	2.5	3.0	3.4	0.1	---
1001	1002	α-Phellandrene	---	0.4	0.3	0.8	---	---
1007	1008	δ-3-Carene	---	14.3	9.3	4.5	---	---
1012	1014	α-Terpinene	0.0	---	---	---	0.7	1.4
1018	1020	*p*-Cymene	---	0.1	---	---	---	---
1025	1024	Limonene	38.1	31.1	36.5	30.8	29.7	24.3
1042	1044	(*E*)-β-Ocimene	tr ^c^	tr	tr	0.1	---	0.3
1054	1054	γ-Terpinene	0.5	0.1	0.2	0.7	1.1	2.0
1061	1065	*cis*-Sabinene hydrate	0.2	---	0.1	0.6	0.4	0.8
1084	1086	Terpinolene	0.3	0.8	0.5	0.4	0.2	0.4
1094	1095	Linalool	0.6	1.6	1.1	3.4	1.7	1.2
1116	1118	*cis*-*p*-Menth-2-en-1-ol	0.1	---	tr	0.2	0.2	0.3
1133	1136	*trans-p*-Menth-2-en-1-ol	0.1	---	---	0.1	0.1	0.1
1174	1174	Terpinen-4-ol	2.3	0.1	0.8	2.9	4.2	5.6
1186	1186	α-Terpineol	0.5	0.3	0.4	0.2	0.2	0.2
1196	1194	Myrtenol	---	0.1	---	---	---	---
1208	1214	Linalyl formate	---	0.1	---	---	---	---
1222	1227	Nerol	0.1	---	---	0.1	---	---
1334	1335	δ-Elemene	tr	0.4	tr	0.1	---	---
1344	1346	α-Terpinyl acetate	0.1	---	---	---	---	---
1373	1374	α-Copaene	0.4	0.6	---	---	---	---
1389	1389	β-Elemene	0.1	0.4	0.4	0.4	---	tr
1410	1411	*cis*-α-Bergamotene	---	---	---	---	tr	---
1416	1417	(*E*)-β-Caryophyllene	6.9	14.8	6.2	6.3	0.7	2.5
1430	1432	*trans*-α-Bergamotene	---	---	---	---	0.1	---
1431	1434	γ-Elemene	---	---	---	1.2	---	0.2
1450	1454	(*E*)-β-Farnesene	---	---	---	---	tr	---
1452	1452	α-Humulene	0.4	0.9	0.5	0.4	---	0.1
1478	1484	Germacrene D	---	tr	---	0.1	tr	---
1490	1493	α-Zingiberene	---	---	---	---	tr	---
1491	1493	*epi*-Cubebol	0.3	---	---	---	---	---
1492	1489	β-Selinene	0.1	0.5	1.0	0.2	---	0.1
1493	1499	Curzerene	---	---	---	0.6	---	0.2
1505	1505	β-Bisabolene	0.5	0.1	0.2	0.4	0.1	---
1513	1514	Cubebol	0.3	0.1	tr	---	---	---
1521	1522	δ-Cadinene	0.4	0.3	---	---	---	---
1527	1529	(*E*)-γ-Bisabolene	---	---	---	---	tr	---
1546	1548	α-Elemol	---	tr	0.1	0.2	1.6	tr
1554	1559	Germacrene B	---	---	---	0.1	---	---
1562	1561	(*E*)-Nerolidol	---	---	---	1.6	---	---
1577	1577	Spathulenol	---	0.1	---	---	---	---
1582	1582	Caryophyllene oxide	0.5	1.0	0.4	1.6	0.1	0.6
1588	1596	Fokienol	---	0.2	---	---	---	---
1607	1608	Humulene epoxide II	---	---	---	0.3	---	---
1610	1602	Ledol	---	---	tr	---	---	---
1624	1632	α-Acorenol	---	---	---	---	---	0.1
1625	1627	1-*epi*-Cubenol	0.2	---	---	---	---	---
1630	1630	Muurola-4,10(14)-dien-1β-ol	---	1.1	---	0.8	---	---
1636	1639	Caryophylla-4(12),8(13)-dien-5β-ol	---	0.2	---	---	---	---
1640	1640	τ-Murrolol	0.3	---	---	---	---	---
1644	1644	α-Muurolol (=δ-Cadinol)	1.3	tr	tr	---	---	---
1649	1649	β-Eudesmol	---	---	---	---	0.1	---
1656	1658	*neo*-Intermedeol	---	tr	0.2	---	---	---
1668	1668	14-Hydroxy-9-*epi*-(*E*)-caryophyllene	---	0.1	---	0.1	---	---
1677	1679	Khusinol	---	0.2	---	---	---	---
1682	1685	α-Bisabolol	---	tr	---	---	1.1	---
1714	1713	14-Hydroxy-α-humulene	---	---	---	0.2	---	---
1727	1728	*iso*-Longifolol	---	tr	---	---	---	---
1763	1762	β-Acoradienol	---	---	---	0.3	---	---
1930	1929	Musk ambrette	---	0.2	---	---	---	---
		Monoterpene hydrocarbons	84.4	76.6	87.6	77.9	89.5	88.0
		Oxygenated monoterpenoids	3.8	2.2	2.4	7.3	6.7	8.2
		Sesquiterpene hydrocarbons	8.9	17.8	8.3	9.7	0.8	3.0
		Oxygenated sesquiterpenoids	2.9	3.0	0.6	5.0	2.9	0.7
		Total identified	99.9	99.5	99.0	99.9	99.8	99.9

^a^ RI = “Retention Index” determined in reference to a homologous series of *n*-alkanes. ^b^ Retention indices from the databases [22,23]. ^c^ tr = “trace” (<0.05%).

**Table 3:** Chemical compositions (major components, %) of *Piper nigrum* volatile oils reported in the literature.

Compound	α-Thujene	α-Pinene	Camphene	Sabinene	β-Pinene	Myrcene	α-Phellandrene	δ-3-Carene	*p*-Cymene	Limonene	β-Phellandrene	(*E*)-β-Ocimene	Terpinolene	Linalool	δ-Elemene	α-Cubebene	α-Copaene	β-Elemene	β-Caryophyllene	α-Guaiene	α-Humulene	Germacrene D	β-Selinene	α-Selinene	β-Bisabolene	α-Farnesene	δ-Cadinene	Elemol	Caryophyllene oxide
Balankotta [29]	0	20.9	0	0	0	13.5	0	11.7	8.2	25.2	0	0	0	0	0	0	0	0	7.7	0	0	0	0	0	0	0	0	0	0
Brazil, CO_2_ [20]	1	4.1	0	11.6	2.6	7.7	1.9	14.4	1.8	19.8	0.4	0	0.7	0.4	0.8	0	1.3	1.3	21.8	0	1.5	0	2.5	3.1	0	0	0.5	0	0
Subh-90 [26]	0.1	7	0.2	0.5	7.6	7.9	0.1	19	2.3	22.7	0	0	0.1	0.5	0	0.1	0.9	0.2	7.6	0.1	0.3	0	0.1	tr	1.6	0	0.1	0.7	6
Subh-91 [26]	0.1	3.2	0.2	0.2	8	6.7	3.5	23.4	0.9	19.5	0	0.1	0	0.6	0	0.3	1.7	0.2	15.5	0	0.4	0	0.1	0.1	2.8	0	0	0.8	0.4
Subh-92 [26]	0.1	4.7	0.1	0.2	9.6	4.3	3.8	20.8	0.6	18.3	0	0	0	0.5	0	0.1	1.7	0.1	21.3	0	0.4	0	0.1	0.1	3.1	0	0	0.8	3
Cameroon [1]	1.8	5.6	0.1	11.2	6.7	2.5	4.5	18.5	0.7	14.7	0	0.1	1.2	0.7	1.7	0.2	1.4	1.3	12.8	0	1.3	0.2	0	2.2	tr	0	0.6	0	0
Sree-III [27]	tr	4.3	0.2	0.2	10.2	5.5	3	11.1	0.5	20.1	0	0.1	0.1	0.2	0	tr	1.5	0.1	23.1	0	0.4	0	tr	tr	2.5	0	0	0.6	0.3
Uthir-1st [28]	0.2	14.6	0.4	0.3	9.3	4.3	7.4	8.5	1.3	19.5	0	0	0	0.1	0	tr	0.9	0.2	25.1	0	tr	0	tr	0	0	0	0.1	tr	0.6
Kari-90 [30]	0.1	5.4	0.2	0.2	15.2	0	3.3	20.3	0.7	20.1	0	0	0	0.5	0	1.9	0	0.1	19.8	0	0.4	0	0.1	0.1	2.5	0	0.1	0.8	0.4
Kari-91 [30]	tr	5	0.1	0	14.3	0.8	2.8	21	0.6	19.7	0	tr	0	tr	0	2.2	0	0	20.6	0	tr	0	0.1	0.1	2.9	0	0.2	0.9	0.4
Kari-93 [30]	0.1	5.3	0.1	0.6	14.1	0.9	2.9	17.8	0.9	19.6	0	0.2	0.2	0.5	0	1.5	0	0.1	25.6	0	0.4	0	0.1	0.1	2.7	0	0	0.8	0.5
Madagascar [24]	0	25.4	0.8	0	15.7	0	0	10.8	1	21	0	0	0	0.6	1.5	0	0	0	0	0	0	0	0	0	0	0	0	0	0
Thom-90 [29]	0.8	12.9	0.3	3.8	6.4	6.3	2.2	12.6	0.6	16.4	0	0.3	0.1	0.6	0	1.3	0.9	0	23.5	0	tr	0	0.1	0.3	0	0	tr	0	0.8
Market [29]	2.4	10	0	0	24.4	15.2	0	0	0	26.5	0	0	0	0	0	3.5	0	0	2.4	0	0	0	0	0	0	4.6	0	0	6.2
Pann-1 [29]	3	7.7	0	0	21.2	13.8	1.3	3.4	0	21.1	0	0	0	0	0	2.2	0	0	10.6	0	0	0	0	0	0	5.9	0	0	0
Pann-5 [29]	2.8	7.1	0	0	22.3	12.3	0	2.3	0	20.3	0	0	0	0	0	2	0	0	17.8	0	0	0	0	0	0	16.7	0	0	0
Aimp-3rd [28]	2.3	6.6	0.2	0	23.9	11.1	0.4	0	0.2	21	0	0.1	0.1	0.2	0	tr	0.4	1	16.4	0	1.1	0	0.3	0.7	0	0	0	9.6	1.2
Orav 2004 [3]	0.2	7.3	0.2	1.4	19	2.6	2.2	10.6	0.5	29.7	0.3	0	0.6	2.1	0	1.6	0	0	14	0	0	tr	0.3	0.3	0.2	0.2	0.6	0.4	0.8
Bagheri 2014 [5]	1.4	8	0.3	13.2	9.7	1.2	1.6	8.6	0.9	15	0	0	0.2	0.6															
Thev-90 [26]	1.1	3.8	tr	10.5	8.3	0	0.7	5.3	0.3	13.7	0	tr	0.1	0.4															
Thev-91 [26]	1.2	5.2	tr	16.2	8.7	0	0.6	5.5	0.5	18	0	0.2	0.1	0.2															
Thev-92 [26]	0.5	1.9	tr	4.5	3.7	1.6	0.9	4.8	0.2	8.3	0	0.1	tr	0.3															
Poon-90 [26]	1.5	5.1	0.2	4.5	11.7	6.6	1.4	2.1	0.4	15.8	0	12	0.1	0.8															
Poon-91 [26]	0.8	4.9	0.1	2.3	10.2	7.2	1.2	2.1	6.2	15.2	0	0.2	0.1	0.5															
Poon-92 [26]	0.8	3	0.1	7.8	6	4.1	1.5	7.3	0	14.9	0	tr	0.1	0.5															
Valia-90 [26]	1.1	6.3	0.3	17.1	0	0.2	0.7	0	0.7	18.6	0	0	0.1	0.1															
Valia-91 [26]	1.1	4.6	0.4	15.9	tr	0.2	2.1	10.5	0.3	15.9	0	tr	0.2	0.1															
Valiai-92 [26]	0.8	2.9	0.3	12.9	0	0.1	1.6	8.7	0	12.9	0	0	0.1	0.1															
S.Tome e Principe [25]	1.4	5.7	0.1	16.5	10.7	2	0.7	1.7	0.2	18.8	2.9	0.5	0.4	1.1															
Market (India) [2]	0.8	4.5	0.1	5.9	7.9	1	0.6	4.4	1.2	13.2	0	tr	0.1	0.5															
Sree-I [27]	tr	5.5	0.2	4.3	11.2	0	7.7	0.1	1.5	22.1	0	tr	0	0.5															
Sree-II [27]	1.5	3.3	0.1	4.6	0	9.6	0	0.1	1.5	20.5	0	0.2	0.2	0.6															
Kuch-I [27]	tr	5.4	tr	13.3	0	0	0	0.4	0	14.5	0	0	0.1	0.4															
Kuch-II [27]	0.7	2.3	0.1	6.7	5.2	5.2	6.2	0.5	2	17.5	0	0.2	0.1	0.4															
Vell-I [27]	1.7	3.6	0.2	18.8	10.9	0	0.2	tr	0.2	19.8	0	0	0.1	0.4															
Vell-II [27]	1	3.6	0.1	8.4	6.5	3.1	1.3	7.6	0.3	14.9	0	0.1	0.1	0.5															
Vell-III [27]	0.4	1.7	0.1	3.9	3.9	2	1	5.1	0.1	8.3	0	tr	tr	0.3															
Aimp-1st [28]	0.9	8.4	tr	27.5	9.2	0	tr	0.1	0.5	19.8	0	tr	0.3	0.2	0	tr													
Aimp-2nd [28]	0.5	7.4	0.2	24.2	14.8	0	0.2	0	0.3	22.5	0	0.2	0.2	0.5	0	tr													
Naray-1st [28]	2.7	5.9	tr	24.6	8.7	0	0.3	2.3	0.4	15.5	0	0.1	0.1	0.5	0	tr													
Naray-2nd [28]	0.3	6.4	tr	4.4	15.6	8.4	0.1	0	0.3	19.5	0	0.2	0.1	0.2	0	tr													
Naray-3rd [28]	1	2	0.1	13.9	4.8	0	0.2	tr	0.2	9.5	0	tr	0.2	0.1	0	tr													
Neel-1st [28]	1.6	6.5	0.2	27.3	11.3	0	1.3	7.9	0.5	18.6	0	0	0.1	0.6	0	0.2													
Neel-2nd [28]	1	5.6	0.2	23.9	7.8	0	0.4	0.5	0.3	15.9	0	tr	0.2	0.3	0	0.7													
Neel-3rd [28]	2.2	4.7	tr	23.2	9.8	0.3	0.4	0.1	0.1	12.9	0	0	0.3	0.3	0	0.1													
Uthir-2nd [28]	0.1	9.1	0.2	0.1	12.5	3.5	5	6.7	1.1	13.3	0	0.1	0	0.1	0	0.2													
Kott-90 [31]	2.5	7.4	0.1	18.8	15.4	0	0.3	0.2	0.2	23.8	0	0.2	0.3	0.5	0	0.8													
Kott-91 [31]	2.4	7.1	0.1	22.1	13.3	0	0.2	0.2	0.2	21.5	0	0.2	0.2	0.5	0	0.1													
Kott-92 [31]	1	3	tr	11.2	7.5	0	0.2	tr	0.1	12.7	0	0.3	0.2	0.1	0	0.2													
Ott-90 [31]	0.7	4.4	0.1	9.1	3.8	8.3	0.1	tr	0.1	15.5	0	0.2	0.1	0.1	0	0.1													
Ott-91 [31]	2	5.9	0.1	26.8	11	0	0.4	0.1	0.4	20.2	0	0.5	0.2	0.3	0	0.2													
Ott-92 [31]	0.6	1.8	0.1	0.1	11.7	18.6	0.2	tr	0.4	21.7	0	0.4	0.2	0.3	0	0.2													
Kuth-90 [31]	0.6	7.9	0.3	5.3	10.9	0.2	6.8	0	1	16.9	0	0	0.1	1.2	0	0.2													
Kuth-92 [31]	0.2	2.7	0.1	1.9	3.8	2	0.5	4.2	0.3	9	0	0.1	tr	0.7	0	0.3													
Cher-90 [31]	0.1	7.1	0.2	9.7	11.2	3	1.2	3.2	4	17.8	0	0.4	0.1	0.3	0	0.1	0.3	0.1											
Cher-91 [31]	3.6	4	0.1	22.3	7.7	0	2.6	5.4	1.5	15.2	0	0.5	0.2	0.3	0	2.5	0.9	0.1											
Cher-92 [31]	2	5	0.1	19.1	9.5	0.6	2.2	9.8	0.4	14.7	0	0.5	0.2	0.6	0	2.9	0.9	0.1											
Kall-90 [30]	tr	10.1	0.4	8	11.7	0	9.8	0.9	0	18.1	0	0.1	0.1	1.3	0	0.8	0	0.1											
Kall-91 [30]	0.4	5.8	0.2	7.1	8.1	0	6.1	3.7	0.9	19.1	0	0.2	0.1	1.3	0	0.8	0.1	0											
Kall-93 [30]	0.2	3.5	0.1	1.8	4.4	2.6	0.4	4.5	0.3	10.7	0	0.2	tr	0.7	0	1.3	0.1	0.1											
Thom-91 [30]	0.1	2.4	0.1	3.9	2	4	0.5	2.5	0.7	11.7	0	0	0.1	0.5	0	3.8	0.9	0											
Thom-93 [30]	0.7	11.8	0.3	4.1	7.3	0.4	0.9	5.4	0.4	9.4	0	0.2	0.1	0.4	0	3	0.7	0											
Arak-90 [30]	2.2	6.9	0.1	20.9	11.1	0	0.3	0.2	0	20.4	0	0.4	0.2	0.3	0	0.1	0.7	tr											
Arak-91 [30]	0.4	4.4	0.6	5.5	12.5	2.4	0.2	0.1	0.2	21.9	0	0.4	0.1	0.1	0	0.1	0.6	1											
Arak-93 [30]	0.6	2.4	tr	6.3	6.1	0.2	0.1	0.4	0.1	10.3	0	0.2	0.1	0.2	0	0.6	1.1	tr											

**Table 4:** Concentration (%) of centroids used in the cluster analysis of *Piper nigrum* oils.

Compound	Cluster 1	Cluster 2	Cluster 3	Cluster 4
α-Thujene	0.610	1.510	0.323	1.250
α-Pinene	4.538	6.089	10.608	8.337
Camphene	0.173	0.125	0.288	0.036
Sabinene	5.892	19.141	2.179	0.175
β-Pinene	6.982	9.621	10.004	28.796
Myrcene	3.512	0.386	4.164	6.040
α-Phellandrene	2.169	0.688	2.686	0.485
δ-3-Carene	3.146	2.812	14.998	4.031
*p*-Cymene	0.758	0.546	1.324	0.074
Limonene	14.850	17.499	19.404	28.103
β-Phellandrene	0.000	0.173	0.111	0.027
(*E*)-β-Ocimene	0.588	0.190	0.111	0.053
Terpinolene	0.097	0.184	0.272	0.302
Linalool	0.473	0.398	0.412	1.083
δ-Elemene	0.019	0.223	0.675	0.049
α-Cubebene	1.661	0.777	0.477	0.844
α-Copaene	0.947	0.777	0.814	0.132
β-Elemene	0.148	0.402	0.515	0.205
β-Caryophyllene	33.815	20.759	17.184	8.951
α-Guaiene	0.183	0.202	0.088	0.000
α-Humulene	0.272	0.523	0.635	0.299
Germacrene D	0.012	0.015	0.465	0.014
β-Selinene	0.206	0.295	0.593	0.223
α-Selinene	0.483	0.500	0.674	0.091
α-Farnesene	0.000	0.000	1.088	2.495
β-Bisabolene	1.485	1.147	0.005	0.135
δ-Cadinene	0.134	0.381	0.165	0.120
Elemol	1.609	1.595	0.321	1.080
Caryophyllene oxide	1.923	1.182	0.863	1.135

## References

[1] Tchoumbougnang F., Dongmo P.M.J., Sameza M.L., Fombotioh N., Wouatsa N.A.V., Amvam Z.P.H., Menut C. (2009). Comparative essential oils composition and insecticidal effect of different tissues of *Piper capense* L., *Piper guineense* Schum. et Thonn., *Piper nigrum* L. and *Piper umbellatum* L. grown in Cameroon. Afr. J. Biotechnol..

[2] Kapoor I.P.S., Singh B., Singh G., De Heluani C.S., De Lampasona M.P., Catalan C.A.N. (2009). Chemistry and in vitro antioxidant activity of volatile oil and oleoresins of black pepper (*Piper nigrum*). J. Agric. Food Chem..

[3] Orav A., Stulova I., Kailas T., Müürisepp M. (2004). Effect of storage on the essential oil composition of *Piper nigrum* L. fruits of different ripening states. J. Agric. Food Chem..

[4] Salehi B., Zakaria Z.A., Gyawali R., Ibrahim S.A., Rajkovic J., Shinwari Z.K., Khan T., Sharifi-Rad J., Ozleyen A., Turkdonmez E. (2019). *Piper* species: A comprehensive review on their phytochemistry, biological activities and applications. Molecules.

[5] Bagheri H., Bin Abdul Manap M.Y., Solati Z. (2014). Antioxidant activity of *Piper nigrum* L. essential oil extracted by supercritical CO_2_ extraction and hydro-distillation. Talanta.

[6] Ramnik S., Narinder S., Saini B.S., Harwinder S.R. (2008). In vitro antioxidant activity of pet ether extract of black pepper. Ind. J. Pharmacol..

[7] Singh G., Maurya S., Catalan C., De Lampasona M.P. (2004). Chemical, antioxidant and antifungal activities of volatile oil of black pepper and its acetone extract. J. Sci. Food Agric..

[8] Jeena K., Liju V.B., Umadevi N.P., Kuttan R. (2014). Antioxidant, anti-inflammtory and antinociceptive properties of black pepper essential oil (*Piper nigrum* Linn). J. Essent. Oil Bear. Plants.

[9] Dorman H.J.D., Deans S.G. (2000). Antimicrobial agents from plants: Antibacterial activity of plant volatile oils. J. Appl. Microbiol..

[10] Lee J., Lee J.H., Kim S.I., Cho M.H., Lee J. (2014). Anti-biofilm, anti-hemolysis, and anti-virulence activities of black pepper, cananga, myrrh oils, and nerolidol against *Staphylococcus aureus*. Appl. Microbiol. Biotechnol..

[11] Hashim S., Aboobaker V.S., Madhubala R., Bhattacharya R.K., Rao A.R. (1994). Modulatory effects of essential oils from spices on the formation of DNA adduct by aflatoxin B1 in vitro. Nutr. Cancer.

[12] Kristiniak S., Harpel J., Breckenridge D.M., Buckle J. (2012). Black pepper essential oil to enhance intravenous catheter insertion in patients with poor vein visibility. J. Altern. Complement. Med..

[13] Ebihara T., Ebihara Ã.S., Maruyama Ã.M., Kobayashi M., Itou A., Arai H., Sasaki H. (2006). A Randomized trial of olfactory stimulation using black pepper oil in older people with swallowing dysfunction. J. Am. Geriatr. Soc..

[14] Rose J.E., Behm F.M. (1994). Inhalation of vapor from black pepper extract reduces smoking withdrawal symptoms. Drug Alcohol Depend..

[15] Cordell B., Buckle J. (2013). The effects of aromatherapy on nicotine craving on a U.S. campus: A small comparison study. J. Altern. Complement. Med..

[16] Mori M., Ikeda N., Kato Y., Minamino M., Watabe K. (2003). Inhibition of elastase activity by essential oils in vitro. J. Cosmet. Dermatol..

[17] Liu Y., Yadev V.R., Aggarwal B.B., Nair M.G. (2010). Inhibitory effects of black pepper (*Piper nigrum*) extracts and compounds on human tumor cell proliferation, cyclooxygenase enzymes, lipid peroxidation and nuclear transcription factor-kappa-B. Nat. Prod. Commun..

[18] Costa R., Machado J., Abreu C. (2016). Evaluation of analgesic properties of *Piper nigrum* essential oil: A randomized, double-blind, placebo-controlled study. World J. Tradit. Chin. Med..

[19] Liu L., Song G., Hu Y. (2007). GC-MS analysis of the essential oils of *Piper nigrum* L. and *Piper longum* L.. Chromatographia.

[20] Ferreira S.R.S., Nikolov Z.L., Doraiswamy L.K., Meireles M.A.A., Petenate A.J. (1999). Supercritical fluid extraction of black pepper (*Piper nigrum* L.) essential oil. J. Supercrit. Fluids.

[21] Zacharia T.J., Gopalam A. (1987). Nature, production and quality of essential oils of pepper, ginger, turmeric, cardamom and tree spices. Indian Perfum..

[22] Adams R.P. (2007). Identification of Essential Oil Components by Gas. Chromatography/Mass Spectrometry.

[23] Satyal P. (2015). Development of GC-MS Database of Essential Oil Components by the Analysis of Natural Essential Oils and Synthetic Compounds and Discovery of Biologically Active Novel Chemotypes in Essential Oils. Ph.D. Thesis.

[24] Möllenbeck S., König T., Schreier P., Schwab W., Rajaonarivony Ã.J., Ranarivelo L. (1997). Chemical composition and analyses of enantiomers of essential oils from Madagascar. Flav. Fragr. J..

[25] Martins A.P., Salgueiro L., Vila R., Tomi F., Cañigueral S., Casanova J., Proença Da Cunha A., Adzet T. (1998). Essential oils from four *Piper* species. Phytochemistry.

[26] Nirmala Menon A., Padmakumari K.P., Jayalekshmy A. (2003). Essential oil composition of four major cultivars of black pepper (*Piper nigrum* L.) III. J. Essent. Oil Res..

[27] Nirmala Menon A., Padmakumari K.P. (2005). Studies on essential oil composition of cultivars of black pepper (*Piper nigrum* L.)-V. J. Essent. Oil Res..

[28] Nirmala Menon A., Padmakumari K.P. (2005). Essential oil composition of four major cultivars of black pepper (*Piper nigrum* L.)-IV. J. Essent. Oil Res..

[29] Mamatha B.S., Prakash M., Nagarajan S., Bhat K.K. (2008). Evaluation of the flavor quality of pepper (*Piper nigrum* L.) cultivars by GC-MS, electronic nose and sensory analysis techniques. J. Sens. Stud..

[30] Nirmala Menon A., Padmakumari K.P., Jayalekshmy A., Gopalakrishnan M., Narayanan C.S. (2000). Essential oil composition of four popular Indian cultivars of black pepper (*Piper nigrum* L.). J. Essent. Oil Res..

[31] Menon A.N., Padmakumari K.P., Jayalekshmy A. (2002). Essential oil composition of cultivars of four major cultivars of black pepper (*Piper nigrum* L.). J. Essent. Oil Res..

